# The Prevalence, Predictors, and In-Hospital Outcomes of Stroke-Associated Infection in Acute Ischemic Stroke: A Malaysian Prospective Cohort Study

**DOI:** 10.3390/jcm15134898

**Published:** 2026-06-24

**Authors:** Shausha Mohamed Anees, Xiong Khee Cheong, Hui Jan Tan, Najma Kori, Wan Nur Nafisah Wan Yahya, Rosnah Sutan, Petrick Periyasamy

**Affiliations:** 1 Infectious Disease Unit, Department of Medicine, Faculty of Medicine, Universiti Kebangsaan Malaysia, Cheras 56000, Malaysia; shauz.anys@hotmail.com (S.M.A.);; 2 Neurology Unit, Department of Medicine, Faculty of Medicine, Universiti Kebangsaan Malaysia, Cheras 56000, Malaysia; 3 Department of Public Health, Faculty of Medicine, Universiti Kebangsaan Malaysia, Cheras 56000, Malaysia

**Keywords:** stroke-associated infection, pneumonia, acute ischemic stroke, NIHSS, modified Rankin Scale, functional outcome

## Abstract

**Background:** Stroke-associated infection (SAI) is a common complication of acute ischemic stroke and is associated with adverse clinical outcomes. Contemporary prospective data from Southeast Asia remain limited. The primary objective was to determine the prevalence of SAI in patients with acute ischemic stroke. Secondary objectives were to identify associated clinical predictors and evaluate its relationship with in-hospital outcomes. **Methods:** This prospective observational cohort study included 390 adults with acute ischemic stroke admitted to a tertiary center between August 2024 and November 2025. SAI was defined as clinically diagnosed infection occurring within seven days of stroke onset using standardized criteria. Demographic, clinical, and treatment variables were collected. Stroke severity was assessed using the National Institutes of Health Stroke Scale (NIHSS). Functional outcome at discharge was evaluated using the modified Rankin Scale (mRS). Multivariable logistic regression was performed to identify independent predictors of SAI. **Results:** SAI occurred in 75 patients, giving a prevalence of 19.2% (95% CI 15.3–23.1). Pneumonia was the predominant subtype (14.4%). On multivariable analysis, higher NIHSS score (adjusted OR 1.10 per point; 95% CI 1.05–1.14; *p* < 0.001) and mechanical thrombectomy (adjusted OR 3.02; 95% CI 1.11–8.26; *p* = 0.031) were independently associated with SAI. Patients with SAI had longer hospital stays (median 8 vs. 4 days, *p* < 0.001), poorer functional outcomes (81.3% vs. 24.8% with mRS 3–6, *p* < 0.001), and higher in-hospital mortality (17.3% vs. 1.0%, *p* < 0.001). **Conclusions:** Stroke-associated infection affected approximately one-fifth of patients with acute ischemic stroke and was strongly associated with stroke severity and adverse clinical outcomes. These findings support early risk stratification and targeted preventive strategies in acute stroke care.

## 1. Introduction

Stroke is a leading cause of mortality and disability worldwide, accounting for approximately 7 million deaths and substantial disability-adjusted life years annually [1,2]. In Malaysia, stroke is the third leading cause of mortality, following ischemic heart disease and pneumonia [3]. Crucially, acute ischemic stroke is recognized as a highly heterogeneous disease entity. Within clinical trials and observational cohorts, it is clinically vital to systematically differentiate between distinct ischemic stroke subtypes—including cardioembolic stroke, large-artery atherothrombotic infarct, lacunar infarct, infarct of unusual etiology, and essential cryptogenic stroke. This etiological differentiation is essential because stroke subtypes heavily dictate the baseline distribution of patient risk factors, initial neurological severity, therapeutic eligibility, and overall short-term and long-term prognosis [4]. Infections are among the most common complications following acute ischemic stroke and are associated with adverse clinical outcomes [5].

Stroke-associated infection (SAI) refers to infections occurring within the first seven days after stroke onset, a period of heightened vulnerability due to neurological impairment and stroke-induced immune dysregulation [5,6,7]. This definition distinguishes early infections from the broader category of post-stroke infections, which may occur later and are less consistently defined. Reported infection rates vary widely, ranging from 5% to 65% across studies, reflecting differences in patient populations, study design, and diagnostic criteria [1,5,8,9,10]. More recent studies suggest lower prevalence estimates, although heterogeneity remains.

The pathogenesis of SAI is multifactorial. Early infections are commonly related to aspiration due to dysphagia and impaired consciousness, while later infections are associated with immobility, invasive devices, and prolonged hospitalization [11]. In addition, stroke-induced immunodepression contributes to increased susceptibility to infection through dysregulation of both innate and adaptive immune responses [7].

Among SAI, pneumonia is the most frequent and clinically significant subtype, consistently associated with increased mortality, poorer functional outcomes, and prolonged hospital stay [12]. Urinary tract infection is also common and is often related to catheter use and stroke severity [13]. Stroke severity, typically measured using the National Institutes of Health Stroke Scale (NIHSS), has been identified as a predictor of infection across multiple studies [1,13,14,15].

Despite extensive international data, evidence from Southeast Asia, particularly Malaysia, remains limited. Local differences in patient characteristics and healthcare practices may influence both the prevalence and determinants of SAI. This study aimed to determine the prevalence of stroke-associated infection among patients with acute ischemic stroke and to identify associated clinical factors and in-hospital outcomes in a Malaysian tertiary center.

## 2. Materials and Methods

### 2.1. Ethical Consideration

Ethical approval for this study was obtained from the Universiti Kebangsaan Malaysia Research Ethics Committee (approval code: JEP-2023-610) granted on 12 September 2023. Written informed consent was secured from all participants prior to their participation, in accordance with institutional guidelines.

### 2.2. Subjects

A single-center, prospective observational cohort study was conducted using consecutive sampling to recruit participants from August 2024 to November 2025 at Hospital Canselor Tuanku Muhriz (HCTM), Kuala Lumpur, Malaysia. The study population consisted of adult patients admitted with acute ischemic stroke.

Eligible subjects were aged 18 years and above with a diagnosis of acute ischemic stroke confirmed clinically and radiologically. Patients were recruited consecutively upon admission to minimize selection bias.

Exclusion criteria included patients with hemorrhagic stroke, stroke mimics, patients with severe active pulmonary diseases such as lung malignancy, chronic obstructive pulmonary disease and bronchiectasis, patients with pneumonia or other infections, in the recent 3 months prior to the acute stroke and patients who refused to give written informed consent.

### 2.3. Data Collection

Patients were identified through real-time stroke notifications. A consecutive sampling method was employed to recruit eligible patients until the required sample size was achieved.

Informed consent was obtained either directly from the patient or, in cases where the patient lacked decision-making capacity (e.g., more severe strokes), from a legally acceptable representative. All patients underwent standard management as per standard stroke protocol guidelines.

Data were obtained prospectively. Data on clinical history, demographics, comorbidities, smoking, atrial fibrillation, and blood pressure were obtained on presentation. Radiological features including stroke type, side, and vascular territory (MCA, ACA, or PCA) were recorded based on the initial computed tomography (CT) brain imaging performed at presentation. Stroke severity was defined using the National Institutes of Health Stroke Scale (NIHSS; range 0–42) as minor (1–4), moderate (5–15), moderate-to-severe (16–20), and severe (21–42). Interventional modalities (thrombolysis, mechanical thrombectomy) were obtained. Functional outcome with mRS score at discharge was also obtained.

The diagnosis of infection was based on CDC/PISCES criteria [16,17]. Investigations performed for suspected infection included total white cell count, C-reactive protein, chest radiograph, and microbiological cultures (blood, urine, sputum, tracheal aspirate, bronchoalveolar lavage, and pus cultures). Other biochemical markers of infection, such as procalcitonin, erythrocyte sedimentation rate (ESR), and fibrinogen, were not routinely or uniformly ordered as part of the acute stroke admission protocol at this institution and were consequently omitted from the study design to avoid introducing large numbers of missing data artifacts. Furthermore, while standard microbiological cultures were systematically extracted whenever an infection was clinically suspected, immediate empirical antibiotic administration due to hyperacute clinical urgency occasionally masked bacterial isolation yields. This structural reality was tracked as an inherent clinical parameter rather than missing registry data.

All patients with ischemic stroke who were discharged within the 7 days post-stroke, were followed up regarding symptoms of infection.

### 2.4. Definitions

Stroke-Associated Infection (SAI) was defined as any infection occurring within 7 days of acute ischemic stroke onset, diagnosed according to standardized Centers for Disease Control and Prevention (CDC) criteria and the Pneumonia in Stroke Consensus Group criteria for stroke-associated pneumonia [16,17]. Infection subtypes evaluated included Stroke-Associated Pneumonia (SAP), Urinary Tract Infection (UTI), and other infections including skin and soft tissue infection and pressure sore infection [16]. In-hospital mortality was defined as death occurring during the index hospitalization, corresponding to a modified Rankin Scale (mRS) score of 6 at discharge [18]. Stroke severity was assessed using the National Institutes of Health Stroke Scale (NIHSS; range 0–42), with higher scores indicating greater neurological deficit [19]. Dysphagia was identified based on clinical documentation of swallowing impairment following bedside assessment, defined as the presence of cough or choking immediately or within 1 min after ingestion of calibrated volumes of water (5, 10, and 15 mL, each presented in duplicate), or by formal evaluation by speech-language pathology services using The Swallowing Ability and Function Evaluation (SAFE). Initial antibiotic therapy was defined as the first antibiotic administered for treatment of stroke-associated infection.

### 2.5. Data Analysis

Data analysis was conducted using SPSS version 30.0 (IBM Corp., Armonk, NY, USA) in 2024. Categorical variables were summarized as frequencies and percentages, while continuous variables were assessed for normality using visual inspection and the Shapiro–Wilk test. Normally distributed data were presented as mean ± standard deviation and compared between groups using independent samples *t*-tests, with Welch’s correction applied when the assumption of equal variances was violated. Non-normally distributed continuous and ordinal variables were presented as median (interquartile range) and compared using the Mann–Whitney U test. Categorical variables were compared using Pearson’s chi-square test when expected cell counts were adequate, and Fisher’s exact or Fisher–Freeman–Halton exact tests when chi-square assumptions were violated. Multivariable logistic regression was performed to identify independent predictors of stroke-associated infection with results reported as adjusted odds ratios and 95% confidence intervals. A two-tailed *p*-value < 0.05 was considered statistically significant.

## 3. Results

**Figure 1:**
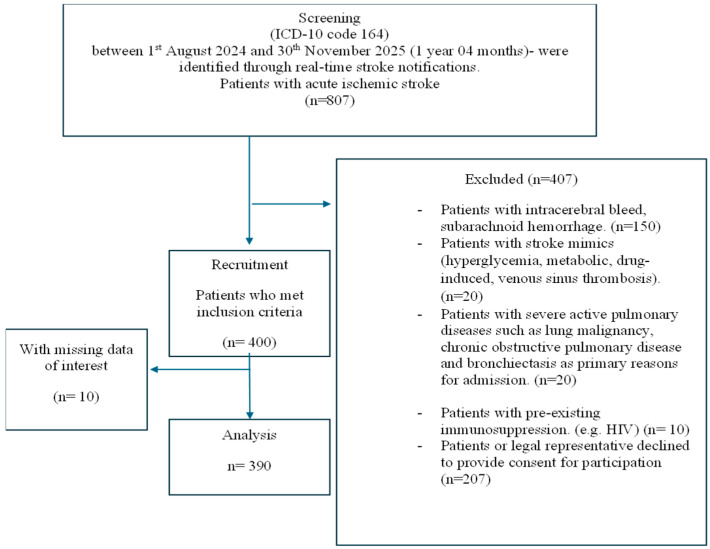
Study flow chart.

A total of 807 patients were identified through real-time stroke notifications (Synapse system and Telegram platform) at HCTM during the study period who met the inclusion criteria. After applying the exclusion criteria and excluding patients with missing data, 390 patients with acute ischemic stroke were included in the final analysis. The study flow chart is shown in Figure 1.

### 3.1. Sociodemographic and Clinical Characteristics

Of the 390 patients included in this study, 75 developed stroke-associated infection (SAI), while 315 did not, as shown in Table 1. On bivariate analysis, patients who developed SAI were significantly older, with a mean age of 67.3 ± 15.2 years compared to 63.4 ± 12.5 years in those without infection (*p* = 0.043). Stroke severity on admission was also significantly higher in the SAI group, with a median NIHSS score of 10 (IQR 6–20) compared to 3 (IQR 1–6) among patients without infection (*p* < 0.001).

Hypertension was the most common comorbidity in both groups and was not significantly associated with SAI (77.3% vs. 78.4%, *p* = 0.839). However, several comorbid conditions were significant among patients who developed SAI, including atrial fibrillation (28.0% vs. 7.9%, *p* < 0.001), ischemic heart disease (30.7% vs. 15.6%, *p* = 0.002), and chronic kidney disease (16.0% vs. 7.0%, *p* = 0.013). Dysphagia was also significantly more common in the SAI group (29.3% vs. 8.3%, *p* < 0.001). Among the 390 patients, 48 had dysphagia, and all required nasogastric Ryle’s tube (RT) insertion. Overall, RT was inserted in 80 patients, indicating that although all patients with dysphagia required RT, additional patients without documented dysphagia also underwent RT insertion, likely reflecting alternative clinical indications such as reduced level of consciousness, inability to maintain adequate oral intake, or need for medication administration. In terms of acute stroke treatment, patients who developed SAI were significantly more likely to receive intravenous thrombolysis (57.3% vs. 22.9%, *p* < 0.001) and mechanical thrombectomy (17.3% vs. 3.2%, *p* < 0.001).

Sex, ethnicity, smoking status, diabetes mellitus, and dyslipidemia were not significantly associated with stroke-associated infection. Biochemical parameters were also comparable between groups, with no significant differences in HbA1c (median 6.7% [IQR 5.8–7.8] vs. 6.3% [5.7–8.0], *p* = 0.519) or LDL cholesterol levels (median 2.7 mmol/L [IQR 2.1–3.8] vs. 3.2 mmol/L [2.3–4.0], *p* = 0.200).

Continuous values are presented as mean ± standard deviation for normally distributed data and median (interquartile range) for non-normally distributed data. * = Independent *t*-test; # = Pearson Chi-square; ‡= Fisher’s exact test: † = Mann–Whitney U test. LDL = Low-density lipoprotein; IVT = Intravenous thrombolysis; MT = Mechanical thrombectomy; NIHSS = National Institutes of Health Stroke Score; ACA = Anterior cerebral artery; MCA = Middle cerebral artery; PCA = Posterior cerebral artery.

The most common infection was pneumonia (14.4%), followed by other infections (3.5%) and urinary tract infections (1.3%). Overall, 56 patients (14.4%) developed pneumonia, comprising 34 (60.7%) early-onset and 22 (39.3%) late-onset cases. Table 2 illustrates the type of stroke-associated infections and antibiotics usage.

Of the 390 patients, 68 (17.4%) had a urinary catheter (CBD) inserted. Urine culture and sensitivity testing was performed in 26 patients (6.7%), of whom 5 of them (19.2%) had positive culture. Among these, 4 (80.0%) had a catheter in situ at the time of culture; however, these cases did not meet CDC criteria for catheter-associated urinary tract infection (CAUTI) because catheter duration was ≤2 days. Overall, 4 cases (80.0%) were non–catheter-associated urinary tract infections (non-CAUTI), while 1 case (20.0%) met criteria for CAUTI.

Other infections were thrombophlebitis (*n* = 8), acute gastroenteritis (*n* = 2), infective endocarditis (*n* = 1), septic arthritis (*n* = 1), shin abscess (*n* = 1), and infected scalp hematoma (*n* = 1).

Amoxicillin–clavulanate was the most frequently prescribed antibiotic, administered to 61.3% of patients with stroke-associated infection. Piperacillin–tazobactam was used in 21.3% of patients, while meropenem was prescribed in 1.3%. More than one antibiotic was administered in several patients, reflecting escalation or combination therapy.

### 3.2. Outcomes

In bivariate analyses (shown in Table 1), patients with stroke-associated infection (SAI) were older and had higher admission NIHSS scores than those without SAI. SAI was significantly associated with atrial fibrillation, ischemic heart disease, chronic kidney disease, dysphagia, and use of intravenous thrombolysis or mechanical thrombectomy (all *p* < 0.05), while no significant associations were observed with sex, ethnicity, hypertension, diabetes mellitus, dyslipidemia, smoking status, HbA1c, LDL cholesterol, or stroke territory. Variables significant in bivariate analysis were subsequently included in multivariable logistic regression.

The multivariable logistic regression (Table 3) demonstrated that higher NIHSS score on admission and mechanical thrombectomy were independently associated with stroke-associated infection. Each one-point increase in NIHSS score was associated with increased odds of stroke-associated infection (adjusted OR 1.10, 95% CI 1.05–1.14; *p* < 0.001). Additionally, undergoing mechanical thrombectomy was independently associated with a triple-fold increase in the odds of developing SAI (adjusted OR 3.02, 95% CI 1.11–8.26; *p* = 0.031).

Age, atrial fibrillation, dysphagia, and intravenous thrombolysis were not independently associated with stroke-associated infection after multivariable logistic regression. The final model (Table 3) is illustrated above.

Patients with stroke-associated infection had a significantly longer hospital stay compared with those without infection. The median length of stay was 8 days (interquartile range [IQR] 5–16) in the infection group and 4 days (IQR 2–7) in the non-infection group. This difference was statistically significant (*p* < 0.001).

Among the 390 patients included in the study, 251 (64.4%) achieved a good functional outcome at discharge (mRS 0–2), while 139 (35.6%) had a poor functional outcome (mRS 3–6). The median mRS at discharge was 1 ([IQR] 0–4) (Table 4).

Functional outcome at discharge differed significantly between patients with and without stroke-associated infection. Patients with SAI were substantially more likely to have a poor functional outcome compared with those without SAI (61/75 [81.3%] vs. 78/315 [24.8%], *p* < 0.001). In addition, median mRS at discharge was significantly higher in the SAI group than in the non-SAI group (4 [IQR 3–5] vs. 1 [IQR 0–2]; *p* < 0.001) (Table 4).

A total of 16 patients died, including 13 with stroke-associated infection (SAI) and 3 without (Table 5). Among patients with stroke-associated infection who died (*n* = 13), stroke severity based on the National Institutes of Health Stroke Scale (NIHSS; range 0–42) was generally moderate to severe. One patient (7.6%) had minor stroke (NIHSS 1–4), five (38.5%) had moderate stroke (NIHSS 5–15), two (15.4%) had moderate-to-severe stroke (NIHSS 16–20), and five (38.5%) had severe stroke (NIHSS 21–42). Overall, 92.3% of deaths occurred in patients with NIHSS ≥ 5, and 38.5% occurred in patients with severe stroke (NIHSS > 20).

Among patients who died, stroke severity distribution did not differ significantly between those with and without stroke-associated infection (Fisher–Freeman–Halton exact test, *p* = 1.000). Interpretation is limited by the small number of deaths in the non-SAI group.

## 4. Discussion

This study provides important prospective data on the prevalence, predictors, and clinical impact of stroke-associated infection (SAI) among patients with acute ischemic stroke in a tertiary care setting in Malaysia. The observed prevalence of SAI in our cohort was 19.2%, highlighting the substantial burden of early post-stroke infections and reaffirming their clinical significance in acute stroke care. This sub-acute burden is similarly reflected in recent European data, such as the clinical cohort evaluated in Medicina (2024), which underscored a comparable clinical trajectory where early-onset post-stroke infections directly compounded short-term functional disability and extended hospital stays [4]. This prevalence is comparable to that reported by Rinawati et al. (2025) in Indonesia (21.4%) 13 and Vermeij et al. (2009), who observed a post-stroke infection rate of 15% in a prospective cohort [5,8]. However, it is higher than estimates from recent studies, including the meta-analysis by Awere-Duodu et al. (2024), which reported a pooled prevalence of 9.14%, with rates of approximately 8–9% in Asian populations, likely reflecting improvements in contemporary stroke care [9]. Earlier studies have reported higher infection rates, including Westendorp et al. (2011) with approximately 30% prevalence, and prospective cohorts such as Kwan et al. (2013) and Popović et al. (2013), reporting rates of 39% and 47%, respectively [1,6,20]. These higher estimates likely reflect broader and less standardized definitions of post-stroke infection, inclusion of infections occurring beyond the acute phase, and recruitment of more heterogeneous or higher-risk populations. In contrast, the present study applied a standardized definition of SAI within a predefined 7-day period. The relatively higher prevalence observed in our study may also be explained by its prospective design with active surveillance and the tertiary referral setting, which may include patients with more severe strokes and increased infection risk.

In the present study, stroke-associated pneumonia (SAP) was the predominant infection subtype, occurring in 14.4% of patients, with 60.7% early-onset and 39.3% late-onset cases. This prevalence is comparable to that reported by Nor Adina et al. (2012) (15.8%) in a prospective study at our center using active surveillance [10]. In contrast, lower rates have been reported in multicenter and registry-based studies, including Vermeij et al. (2009) (7.5%), and Hoffmann et al. (2012) (7.2%) [5,21]. These differences likely reflect variations in case mix, healthcare exposures (e.g., ICU admission and nasogastric tube use), and diagnostic practices, with registry-based studies potentially underestimating pneumonia due to reliance on routine clinical documentation rather than standardized prospective surveillance.

The prevalence of urinary tract infection (UTI) in this study (1.3%) is comparable to that reported by Rinawati et al. (2025) (0.8%), likely reflecting the use of stricter microbiological definitions [8]. In contrast, higher rates have been reported in contemporary cohorts, such as Jitpratoom et al. (2023) (12.7%) [22], and earlier studies including Westendorp et al., Vermeij et al., and Kwan et al. (30%, 7%, and 10%, respectively) [1,5,6]. These differences may be explained by variations in diagnostic criteria, catheterization practices, and study design. The relatively low prevalence in our study may reflect the use of standardized CDC definitions, restriction to infections within 7 days of stroke onset, and a lower rate of urinary catheterization (17.4%), a key risk factor for UTI. Consistent with this, Stott et al. (2009) demonstrated a strong association between catheterization and UTI [11].

In contrast to Jitpratoom et al., where many UTIs were catheter-associated, most cases in our study were classified as non-catheter-associated, as catheter duration was less than 2 days and did not meet CAUTI criteria [22]. This suggests that while catheterization remains important, non-catheter-associated mechanisms may contribute to early-onset UTI.

Interestingly, diabetes mellitus (DM) did not show a statistically significant difference between the SAI and non-SAI cohorts (61.3% vs. 54.9%, *p* = 0.314), despite the well-established immunocompromised baseline state of diabetic individuals. This suggests that during the hyperacute phase (within 7 days of stroke onset), the massive, systemic immune dysregulation triggered directly by acute cerebral tissue ischemia—known as Stroke-Induced Immunodepression Syndrome (SIDS)—exerts an overwhelming effect that effectively eclipses baseline chronic metabolic immunocompromise. The immediate structural and neurological disruptions of the stroke itself, alongside acute procedural exposures, play a more dominant role in early infection pathogenesis than pre-existing diabetic immunosuppression.

Conversely, chronic kidney disease (CKD) demonstrated a significant baseline association with SAI during bivariate screening (16.0% vs. 7.0%, *p* = 0.013). Patients with CKD are characterized by a persistent state of low-grade chronic inflammation and secondary uremic immune dysfunction. When combined with an acute ischemic event, this pre-existing immunoclimacteric state likely acts synergistically with SIDS, significantly exacerbating susceptibility to early opportunistic nosocomial pathogens.

In addition to determining prevalence, this study systematically evaluated clinical characteristics to identify predictors of SAI. Stroke severity, measured by admission NIHSS, was independently associated with stroke-associated infection, with each one-point increase conferring a 10% higher risk (adjusted OR 1.10, 95% CI 1.05–1.14; *p* < 0.001). This aligns with prior prospective studies, although many used broader post-stroke infection definitions rather than the temporally restricted stroke-associated infection applied here [1,13,14,15]. In the GAIN International Trial, Aslanyan et al. (2003) and Fluck et al. (2024) reported that higher baseline NIHSS independently predicted post-stroke infection, particularly urinary tract infection [14,15]. Similarly, a 2025 Chinese cohort by Li et al. also showed that higher admission NIHSS independently predicted post-stroke UTI [13]. Overall, despite variation in infection definitions and timing, the findings consistently support NIHSS as a robust independent predictor of early stroke-associated infection. This finding reflects the central role of neurological impairment in predisposing patients to infection, through mechanisms such as impaired consciousness, reduced airway protection, and dysphagia.

In our multivariable model, mechanical thrombectomy was identified as an independent statistical predictor of stroke-associated infection (adjusted OR 3.02, 95% CI 1.11–8.26; *p* = 0.031). However, evidence from endovascular therapy cohorts remains highly heterogeneous, with some registries demonstrating an association with pneumonia and others showing no relationship after adjusting for disease severity [23,24]. Therefore, this statistical association must be interpreted with caution and should not be seen as a direct biological cause of infection. Instead, undergoing mechanical thrombectomy likely serves as an acute clinical proxy or surrogate marker for a high intensity of hyperacute care. This includes greater underlying procedural complexity, emergent airway management (such as endotracheal intubation or prolonged conscious sedation), intense post-procedural intensive care unit (ICU) monitoring, and dense early immobility, all of which compound a patient’s vulnerability to early respiratory or nosocomial pathogens. Residual confounding may persist despite multivariable adjustment, particularly given its close alignment with high baseline stroke severity and the small proportion of patients undergoing endovascular therapy (5.9%) in this cohort.

Several comorbidities, including atrial fibrillation, ischemic heart disease, chronic kidney disease, and dysphagia, were associated with stroke-associated infection (SAI) in unadjusted analyses but lost significance after multivariable adjustment. This suggests their effects are largely mediated by stroke severity rather than acting as independent risk factors.

Dysphagia showed a similar pattern, being significant only in bivariate analysis. This aligns with Westendorp et al. (2011), who demonstrated that stroke severity, rather than dysphagia, independently predicts pneumonia risk [1]. The frequent need for nasogastric tube insertion in both dysphagic and non-dysphagic patients further supports its role as a marker of overall neurological impairment rather than a direct causal factor.

Atrial fibrillation was not significant after multivariable logistic regression suggesting its interaction with stroke severity and overall vascular comorbidity burden. This is similar to a study by Wang et al. (2021) and Li Yaming et al. (2022) that observed attenuation of significance of atrial fibrillation after multivariable logistic regression adjusting for stroke severity and severity-related complications [25,26]. Nevertheless, it is highly noteworthy that atrial fibrillation was present in a striking 28.0% of the SAI group compared to just 7.9% in the No-SAI group (*p* < 0.001). This overrepresentation is clinically profound because atrial fibrillation is the primary driver of cardioembolic ischemic strokes. The current neurovascular literature demonstrates that patients with a cardioembolic stroke subtype face a significantly poorer short-term prognosis compared to other cerebrovascular variants, such as lacunar or small-vessel infarcts. Cardioembolic events frequently result in abrupt, large-vessel occlusions with massive infarct volumes, high initial neurological deficits, and a heavily compromised capacity for early recovery, rendering these individuals highly susceptible to acute systemic complications like SAI. This shared collinearity with large infarct size and high baseline NIHSS scores explains why atrial fibrillation lost its independent predictive significance during final multivariable logistic adjustments.

In this cohort, 13 of 16 deaths occurred in patients with SAI, underscoring the strong association between infection and mortality. While immediate causes of death were not formally classified as neurological or non-neurological in the database, clinical trajectories showed distinct drivers between the subgroups. Mortality in the non-SAI cohort (1.0%) was driven by primary neurological failure, whereas deaths in the SAI cohort (17.3%) resulted predominantly from non-neurological infectious complications, specifically severe stroke-associated pneumonia and downstream sepsis. Notably, most deaths among infected patients occurred in those with mild-to-moderate-severe stroke severity (61.5%), while 38.5% had severe strokes. Previous studies have demonstrated a graded relationship between increasing NIHSS and risk of infection, mortality, and poor outcomes [1,5]. Aslanyan et al. identified baseline NIHSS as an independent predictor of infection and mortality, while Hoffmann et al. (2012) incorporated stroke severity into a validated pneumonia prediction model [15,21]. Collectively, these findings across diverse populations suggest that SAI is strongly associated with worse survival, likely mediated by systemic inflammation, aspiration, metabolic stress, and interruption of early rehabilitation.

Collectively, these findings reinforce the importance of early identification of patients at risk of SAI and implementation of appropriate preventive strategies as part of comprehensive stroke care. Preventive measures may include early mobilization, dysphagia screening to reduce aspiration risk, and adherence to infection control practices such as minimizing unnecessary urinary catheterization, which have been associated with reduced risk of hospital-acquired infections in stroke populations.

### 4.1. Strengths and Limitations

The strengths of this study include its prospective design, use of a clearly defined stroke-associated infection framework, and application of standardized measures of stroke severity and functional outcome. Multivariable logistic regression was used to adjust for potential confounding factors. Importantly, this study provides contemporary Malaysian data on stroke-associated infection, addressing a recognized gap in the local literature. In contrast to prior prospective Malaysian studies that focused exclusively on stroke-associated pneumonia in small cohorts of first-ever ischemic stroke patients, the present study examined stroke-associated infection as a broader clinical entity, encompassing pneumonia, urinary tract infection, and other infections within a larger and more heterogeneous stroke population. This approach enabled a more comprehensive assessment of infection burden, discharge functional outcomes, and antibiotic treatment practices, which have not been previously examined in local studies.

Several limitations should be acknowledged. First, this was a single-center study, which may limit the generalizability of the findings to other healthcare settings. Crucially, a key methodological consideration is the high volume of excluded individuals (407 out of 807 screened patients), which prominently includes 207 cases where the patient or their legal representative declined participation consent. This high screening attrition rate introduces a clear potential for selection bias. It is possible that patients with more critical or fatal stroke profiles, or conversely those with very mild symptoms, were disproportionately represented among the non-consented cohort. Consequently, this selection bias must be kept in mind when evaluating our 19.2% prevalence rate, as it may result in an underestimation or overestimation of the true localized burden of stroke-associated infection. Second, infections occurring after hospital discharge were partly identified through telephone follow-up, which may introduce recall bias despite the use of structured questionnaires and verification with medical records. Variability in infection definitions and diagnostic criteria has also been noted in previous studies. For example, earlier cohorts such as Vermeij et al. (2009) [5] used older CDC criteria, which may under-detect early infections, while other registry-based studies have defined pneumonia partly based on antibiotic initiation, potentially increasing the risk of misclassification.

Third, microbiological confirmation was not available for all infections, and many cases were treated empirically. Antibiotic de-escalation and duration of therapy were not evaluated in this study. Similar limitations have been reported in previous stroke infection studies, where microbiological confirmation was often lacking due to early antibiotic administration and difficulties obtaining adequate respiratory specimens.

Fourth, the lack of strict etiological subtyping (e.g., the TOAST classification) represents a minor limitation, as the protocol focused primarily on radiological vascular territories. While atrial fibrillation and MCA territory distribution provided strong indirect insights into cardioembolic risks, future prospective studies would benefit from incorporating formalized etiological criteria to map SAI prevalence across all specific stroke mechanisms more granularly.

Finally, as with most observational studies, causal relationships between risk factors and infection cannot be definitively established despite adjustment using multivariable logistic regression.

### 4.2. Clinical Implications

The findings of this study have important clinical implications for acute ischemic stroke management. Patients with SAI had significantly prolonged hospital stays, poorer functional outcomes, and markedly higher in-hospital mortality. Early identification of patients at high risk of SAI, particularly those with greater neurological impairment, may facilitate timely implementation of targeted preventive measures. As stroke severity was the strongest independent predictor of infection, the routinely used NIHSS can be incorporated into early risk stratification to guide enhanced surveillance of high-risk patients. Additional factors such as atrial fibrillation, diabetes mellitus, and mechanical thrombectomy may further help identify patients requiring closer monitoring, supporting a risk-stratified rather than uniform approach to infection prevention.

In high-risk patients, preventive strategies including early dysphagia screening, aspiration precautions, careful positioning, oral care, and close clinical monitoring may help reduce infection-related complications. Given that pneumonia was the predominant infection subtype, emphasis on aspiration prevention and multidisciplinary stroke unit care, including early involvement of speech and language therapists and nursing staff, remains important.

The observed reliance on empirical antibiotic therapy, with a substantial proportion of culture-positive cases requiring modification to targeted therapy, highlights the importance of balancing prompt treatment with antimicrobial stewardship principles. Strengthening diagnostic pathways, promoting timely microbiological sampling, and implementing structured antibiotic review within 48–72 h may support more individualized antimicrobial management.

Overall, these findings support refinement of local stroke-unit protocols toward earlier severity-based infection risk stratification, targeted surveillance in patients with mechanical thrombectomy, and structured reassessment of empirical antibiotic therapy, while remaining consistent with current guideline-based care.

## 5. Conclusions

Stroke-associated infection was observed in approximately one-fifth of patients admitted with acute ischemic stroke in this Malaysian cohort, with pneumonia representing the predominant infection subtype. Greater stroke severity at presentation, as measured by the National Institutes of Health Stroke Scale, emerged as the strongest independent predictor of stroke-associated infection. In addition, patients who developed infection experienced significantly longer hospital stays, poorer functional outcomes at discharge, and higher in-hospital mortality compared with those without infection.

From a Malaysian perspective, this study provides contemporary local data on the burden and determinants of stroke-associated infection, addressing a significant gap in the existing literature. Strengthening multidisciplinary stroke unit care, reinforcing systematic dysphagia screening and aspiration prevention practices, and reinforcing antimicrobial stewardship practices are vital for future reduction in SAI.

## Figures and Tables

**Table 1:** Bivariate analysis between SAI and risk factors (Demographic and clinical characteristics).

Variables	SAI (*n* = 75)	No SAI (*n* = 315)	*p*-Value
Age, years (Mean ± SD)	67.3 ± 15.2	63.4 ± 12.5	0.043 *
Male	44 (58.7)	213 (67.6)	0.142 ^#^
Female	31 (41.3)	102 (32.4)	
Ethnicity, *n* (%)			0.474 ‡
Malay	34 (45.3)	139 (44.1)	
Chinese	32 (42.7)	140 (44.4)	
Indian	5 (6.7)	29 (9.2)	
Others	4 (5.3)	7 (2.3)	
Comorbidities			
Hypertension	58 (77.3)	247 (78.4)	0.839 ^#^
Diabetes mellitus	46 (61.3)	173 (54.9)	0.314 ^#^
Dyslipidemia	46 (61.3)	205 (65.1)	0.543 ^#^
Atrial fibrillation	21 (28.0)	25 (7.9)	<0.001 ^#^
Ischemic heart disease	23 (30.7)	49 (15.6)	0.002 ^#^
Chronic kidney disease	12 (16.0)	22 (7.0)	0.013 ‡
Smoking	27 (36.0)	146 (46.3)	0.105 ^#^
Biochemical Parameters			
HbA1C, %	6.7 (5.8–7.8)	6.3 (5.7–8.0)	0.519
LDL, mmol/L	2.7 (2.1–3.8)	3.2 (2.3–4.0)	0.200
NIHSS on admission, median (IQR 25th, 75th)	10 (6.20)	3 (1.6)	<0.001 †
Dysphagia, *n* (%)	22 (29.3)	26 (8.3)	<0.001 ^#^
Stroke territory, *n* (%)			0.110 ‡
ACA	0 (0.0)	11 (3.5)	
MCA	64 (85.3)	237 (75.2)	
PCA	11 (14.7)	67 (21.3)	
Acute stroke treatment			
IV thrombolysis, *n* (%)	43 (57.3)	72 (22.9)	<0.001 ‡
Mechanical thrombectomy, *n* (%)	13 (17.3)	10 (3.2)	<0.001 ‡

Continuous values are presented as mean ± standard deviation for normally distributed data and median (interquartile range) for non-normally distributed data. * = Independent *t*-test; ^#^ = Pearson Chi-square; ‡ = Fisher’s exact test: † = Mann–Whitney U test. LDL = Low-density lipoprotein; NIHSS = National Institutes of Health Stroke Score; ACA = Anterior cerebral artery; MCA = Middle cerebral artery; PCA = Posterior cerebral artery.

**Table 2:** Types of infection and antibiotic therapy among patients with stroke-associated infection.

**Type of Infection**	***n* (%) Among SAI (*n* = 75)**	***n* (%) Overall (N = 390)**
Pneumonia	56 (74.6)	56 (14.4)
Urinary tract infection	5 (6.7)	5 (1.3)
Other infections	14 (18.7)	14 (3.5)
**Antibiotic**	***n* (%) Among SAI (*n* = 75)**	***n* (%) Overall (N = 390)**
Amoxicillin–clavulanate	46 (61.3)	46 (11.8)
Piperacillin–tazobactam	16 (21.3)	16 (4.1)
Meropenem	1 (1.3)	1 (0.3)
Other antibiotics	12 (16.1)	12 (3.0)

Antibiotic therapy was classified according to the first antibiotic initiated.

**Table 3:** Multivariable logistic regression analysis of factors associated with stroke-associated infection.

Variables	Adjusted OR	95% CI	*p*-Value
Age (per year)	1.01	0.99–1.03	0.371
NIHSS on admission (per point)	1.10	1.05–1.14	<0.001
Dysphagia	1.41	0.63–3.16	0.397
Atrial fibrillation	2.15	1.00–4.64	0.050
IV thrombolysis	1.69	0.89–3.21	0.109
Mechanical thrombectomy	3.02	1.11–8.26	0.031

OR = odds ratio; CI = confidence interval; NIHSS = National Institutes of Health Stroke Scale; Variables were selected based on clinical relevance and univariate analysis. A *p*-value < 0.05 was considered statistically significant.

**Table 4:** Functional outcome at discharge in patients with SAI vs. non-SAI.

Outcome (mRS at Discharge)	SAI (*n* = 75)	No SAI (*n* = 315)	*p*-Value
Good outcome (mRS 0–2)	14 (18.7)	237 (75.2)	<0.001 †
Poor outcome (mRS 3–6)	61 (81.3)	78 (24.8)	
Median mRS (IQR)	4 (3–5)	1 (0–2)	<0.001 ‡
Total	75 (100.0)	315 (100.0)	

Values are presented as *n* (%). Percentages are calculated within each column. † Chi-square test; ‡ Mann–Whitney U test; mRS = modified Rankin Scale; SAI = stroke-associated infection. Good outcome defined as mRS 0–2; poor functional outcome defined as mRS 3–6.

**Table 5:** In-hospital mortality * and stroke severity among patients with and without stroke-associated infection.

Group	Deaths	Total	Mortality Rate (%)	*p* Value
No infection	3	315	1.0	<0.001
Stroke-associated infection	13	75	17.3	
Overall	16	390	4.1	
Stroke severity among deaths (NIHSS)	Deaths with SAI (*n* = 13)	Deaths without SAI (*n* = 3)	Total deaths (*n* = 16)	*p* = 1.000 **
Mild (1–4)	1 (7.6%)	0 (0.0%)	1 (6.2%)	
Moderate (5–15)	5 (38.5%)	2 (66.7%)	7 (43.8%)	
Moderate–severe (16–20)	2 (15.4%)	0 (0.0%)	2 (12.5%)	
Severe (>20)	5 (38.5%)	1 (33.3%)	6 (37.5%)	

* In-hospital mortality was defined as death during the index admission (mRS score of 6). ** Fisher–Freeman–Halton exact test comparing NIHSS distribution between deaths with and without stroke-associated infection. NIHSS = National Institutes of Health Stroke Scale.

## Data Availability

Data are available from the authors at reasonable request.

## Abbreviations

The following abbreviations are used in this manuscript:

ACA	Anterior Cerebral Artery
AF	Atrial Fibrillation
BAL	Bronchoalveolar Lavage
CKD	Chronic Kidney Disease
CRP	C-Reactive Protein
DALYs	Disability-Adjusted Life Years
DM	Diabetes Mellitus
HCAI	Healthcare-associated infection
HIV	Human Immunodeficiency Virus
HLA-DR	Human Leukocyte Antigen–DR Isotype
ICU	Intensive Care Unit
IHD	Ischemic Heart Disease
IVT	Intravenous Thrombolysis
LDL	Low-Density Lipoprotein
LOS	Length of Stay
MCA	Middle Cerebral Artery
mRS	Modified Rankin Scale
MT	Mechanical Thrombectomy
NIHSS	National Institutes of Health Stroke Scale
PCA	Posterior Cerebral Artery
SAI	Stroke-Associated Infection
SAP	Stroke-Associated Pneumonia
SIDS	Stroke-Induced Immunodepression Syndrome
UTI	Urinary Tract Infection
